# Construction of an HBPL antibacterial coating on a phase-transition lysozyme-modified titanium surface

**DOI:** 10.3389/froh.2025.1615280

**Published:** 2025-06-27

**Authors:** Zhangyi Li, Xiangyu Zhang, Hengyang Yu, Shuai Zhang, Hong Liang

**Affiliations:** ^1^ School and Hospital of Stomatology, Tianjin Medical University, Tianjin, China; ^2^ Department of Stomatology, Tianjin Fifth Central Hospital, Tianjin, China; ^3^ Department of Stomatology, Ecological City Hospital of Tianjin Fifth Central Hospital, Tianjin, China

**Keywords:** implant, surface modified, phase-transited lysozyme, chitosan, hyaluronic acid, hyperbranched poly-L-lysine

## Abstract

**Background:**

In the field of dental implantation, titanium and its alloys serve as primary materials for implants due to their excellent biocompatibility. However, their insufficient antibacterial properties remain a critical limitation. Bacterial adhesion and subsequent biofilm formation on titanium alloy implant surfaces can trigger peri-implant inflammation, potentially leading to severe complications such as implant failure. To address this challenge, we developed a novel surface modification strategy that endows implants with dual functionality of antibacterial activity and enhanced cellular adhesion, thereby proposing a new approach for preventing and managing peri-implantitis.

**Methods:**

A layer-by-layer (LbL) self-assembly technique was employed to construct polyelectrolyte coatings composed of hyperbranched polylysine (HBPL) and hyaluronic acid (HA) on phase-transitioned lysozyme (PTL)-modified titanium surfaces. The surface characteristics were systematically investigated through scanning electron microscopy (SEM) and energy-dispersive x-ray spectroscopy (EDS). Antibacterial efficacy was evaluated by monitoring bacterial viability and morphological alterations. Cytocompatibility assessments and molecular biological investigations were conducted to examine cellular responses and osteogenesis-related gene expression.

**Results:**

A novel polyelectrolyte coating with favorable biocompatibility and antibacterial properties was successfully fabricated on PTL-modified titanium surfaces. This coating demonstrated significant antimicrobial effects while concurrently promoting osteogenic differentiation to a certain extent.

**Conclusion:**

This study presents a dual-functional implant surface coating with combined antibacterial and osteogenic-enhancing capabilities. The developed strategy provides new insights for clinical surface modification of dental implants and offers a promising solution for peri-implantitis prevention and treatment.

## 1 Introduction

Dental implants are clinically utilized for restoring defective dentition caused by pathological conditions or traumatic injuries, serving as artificial tooth roots. Owing to their excellent biocompatibility and physicochemical properties, titanium and its alloys have gained extensive applications in maxillofacial surgery and the field of dental implantation (1). Although the majority of patients demonstrate favorable postoperative recovery, clinical evidence indicates that approximately 10% of implants fail to function properly or even experience failure post-implantation, ultimately resulting in compromized surgical outcomes (2). Clinical studies reveal that approximately 18% of implant cases exhibit aseptic loosening, while bacterial infection-induced inflammatory complications—peri-implantitis and peri-implant mucositis—are observed in as high as 20% of cases (3). This phenomenon primarily stems from the disruption of the delicate microbial equilibrium within the oral cavity following implant placement, thereby predisposing the site to bacterial colonization-associated infections and elevating the risk of implant failure. Such complications not only compromise patients' quality of daily life but may ultimately lead to implant loosening or failure, imposing substantial financial burdens and psychological distress on affected individuals (4).

To address the insufficient antimicrobial efficacy of titanium implants, researchers have directed their efforts toward the rational design and implementation of surface modification technologies, with the dual objectives of enhancing surface-specific antibacterial properties while maintaining intrinsic biocompatibility. Inspired by natural amyloid proteins, pioneering studies have demonstrated the strategic denaturation of functionally tailored proteins [e.g., lactoferrin (5), lysozyme (6, 7), silk fibroin (8), and bovine serum albumin (9)] to achieve surface adhesion on biomaterials. This denaturation-mediated approach serves dual purposes as both a foundational platform for subsequent surface modifications and a novel bioadhesive strategy for dental implant interfaces. The layer-by-layer (LbL) technique, functioning as an eco-friendly drug delivery system with facile fabrication, operates via electrostatic attraction between oppositely charged polyelectrolytes (10). This cyclic deposition process enables the formation of hierarchically ordered multilayered films through precisely controlled self-assembly mechanisms. The construction of a primarily functionalized interface constitutes the cornerstone for both enabling antibacterial efficacy in biomaterials and ensuring robust immobilization of antimicrobial coatings onto implant surfaces.

This study employed a novel amyloid-like assembly modification material—phase-transitioned lysozyme (PTL) functional coating—characterized by cost-effectiveness, wide availability, structural robustness, and environmental benignity. This innovative approach holds critical significance for primary functional interface modification on material surfaces, providing an effective strategy to synergistically enhance the antibacterial performance and biocompatibility of implant materials.

## 2 Materials and methods

### 2.1 Preparation of smooth titanium sheet

Circular titanium discs with diameters of 4, 8, 15, and 20 mm and thicknesses of 1 and 2 mm were mechanically ground and polished using silicon carbide sandpaper (grit size: 800–2,000). Subsequently, the samples underwent sequential ultrasonic cleaning in acetone, absolute ethanol, and deionized water for 15 min per solvent. After drying at 60°C for 1 h in a vacuum oven, all specimens were sterilized via autoclaving at 121°C under 15 psi pressure for 20 min.

### 2.2 Surface phase transformation lysozyme modification of pure titanium

**Figure 1:**
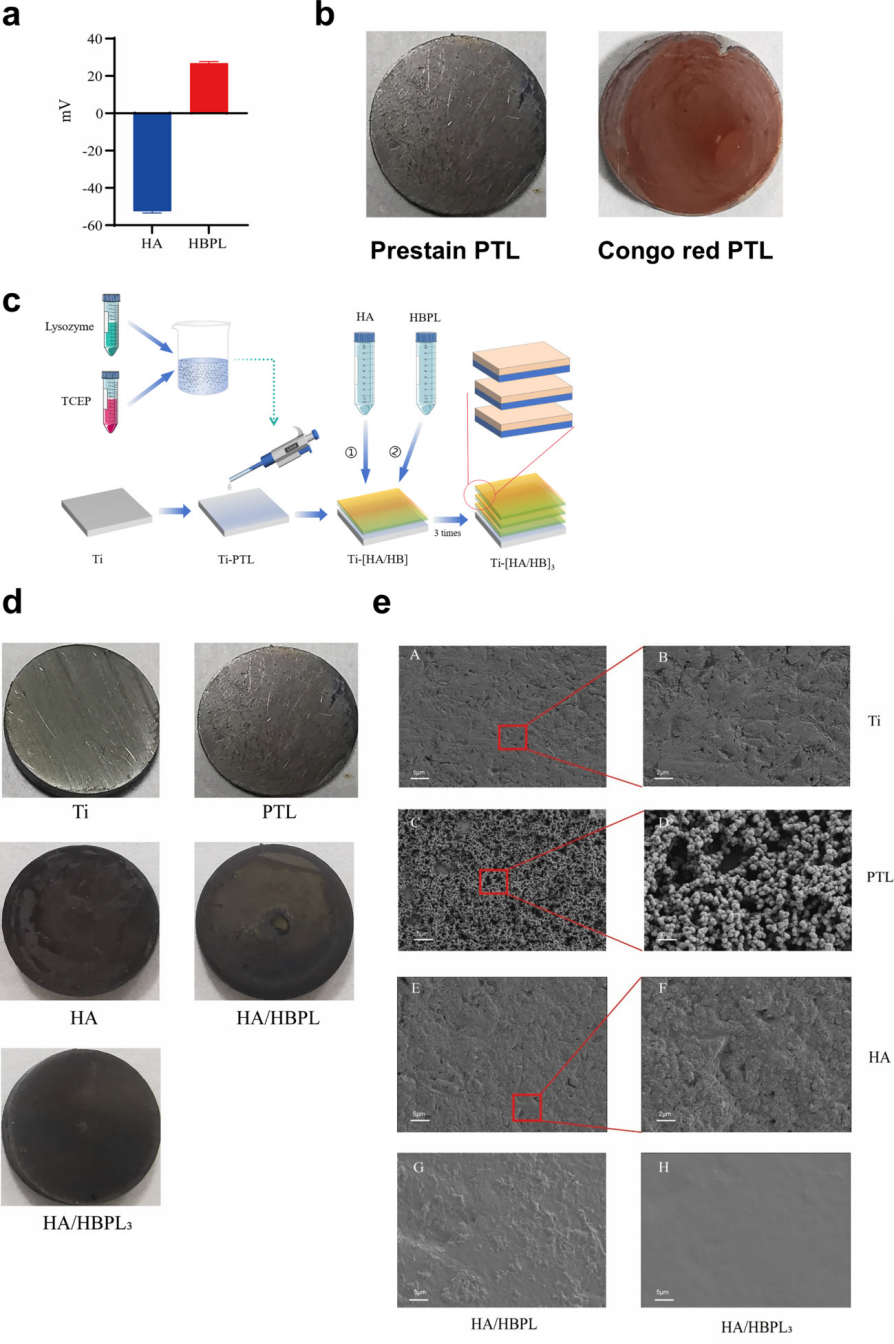
**(a)** Zeta potential values of two polyelectrolytes. **(b)** Congo red staining of titanium discs modified with phase-transitioned lysozyme (PTL). **(c)** Schematic illustration of layer-by-layer (LbL) assembly process for HA/HBPL coating fabrication on PTL-modified titanium. **(d)** Macroscopic photographs of titanium substrates after sequential surface modifications. **(e)** Field-emission scanning electron microscopy (FE-SEM) images of modified specimens post self-assembly.

Lysozyme and TCEP solution were mixed at a 1:1 molar ratio and applied dropwise onto titanium disc surfaces, followed by gentle agitation for homogeneous distribution (11). To mitigate solvent evaporation during incubation, the samples were placed in a humidified chamber containing deionized water and maintained at room temperature for 2 h. Subsequently, the substrates were thoroughly rinsed with HEPES buffer (10 mM, pH 7.4), air-dried under laminar flow, and stored in sterile Petri dishes at 4°C. These functionally modified specimens were designated as Ti-PTL (Figure 1c).

### 2.3 Construction of Layer-by-Layer (LbL) self-assembled polyelectrolyte coatings was performed on modified titanium surfaces

The Ti-PTL samples were immersed in a hyaluronic acid (HA) solution (1 mg/ml) for 30 min, followed by thorough rinsing with 0.14 M sodium chloride (NaCl) solution. After drying under nitrogen flow, the treated substrates were incubated in a hyperbranched polylysine (HBPL) solution (3 mg/ml) for 30 min to form a positively charged polyelectrolyte film, completing one coating assembly cycle (12). The resultant specimens were designated as Ti-PTL-HA/HBPL (abbreviated HA/HB). This cycle was repeated three times to construct multilayered polyelectrolyte composite coatings (Figure 1c), with the final samples labeled Ti-PTL-[HA/HBPL]_3_ [abbreviated (HA/HB)_3_].

### 2.4 Characterization and analysis of titanium substrates with modified polyelectrolyte coatings

Zeta potential measurements of the nanoparticle suspensions were conducted using a Malvern Zetasizer Nano ZS instrument (Malvern Instruments, UK). The prepared electrolyte solutions were adjusted to pH 7.0, and 1 ml aliquots were loaded into disposable zeta potential cuvettes. Measurements were performed at a scattering angle of 173° (backscatter detection mode) with strict temperature control maintained at 25.0 ± 0.1°C using a Peltier thermal regulation system.

Congo red dye, known for its specific binding affinity toward amyloid-like structures, was employed to evaluate protein conformational changes. Titanium specimens were immersed in 1 mg/ml Congo red solution (Sigma-Aldrich) for 10 h under dark conditions. Following incubation, samples were meticulously rinsed with sterile deionized water for 10 h using a peristaltic washing system (50 ml/min flow rate), air-dried in a Class 100 clean bench, and visually inspected for structural coloration.

x-ray photoelectron spectroscopy (XPS, Shimadzu AXIS Supra+, Japan) equipped with a monochromatic Al Kα x-ray source (1,486.6 eV, 15 mA emission current) was utilized to analyze the near-surface chemical composition. All spectra were charge-corrected using adventitious carbon (C 1s = 284.8 eV) as an internal reference. Critical point drying (Leica EM CPD300) was performed prior to sputter-coating samples with a 5 nm gold layer (Quorum Q150T ES, 35 s coating time) to enhance surface conductivity.

Field emission scanning electron microscopy (FE-SEM, ZEISS Sigma 500, Germany) coupled with energy-dispersive x-ray spectroscopy (EDS, Oxford X-MaxN 80) was employed for topographical imaging and elemental mapping at 5 kV accelerating voltage. Atomic force microscopy (AFM, Bruker Dimension Icon, Germany) operated in PeakForce Tapping™ mode (ScanAsyst-Air probes, 0.7 N/m spring constant) provided nanoscale roughness quantification and coating thickness measurements through cross-sectional analysis (*n* = 9 scans per sample).

### 2.5 Contact angle analysis

The hydrophilic properties of modified titanium substrates were evaluated using a dynamic contact angle measurement system (OCA25, Xiamen Chongda Technology Co., Ltd., China) under ambient conditions (25 ± 0.5°C). Deionized water droplets (3 μl) were dispensed onto specimen surfaces via a precision microsyringe. Following droplet stabilization (30 s equilibration time), baseline calibration and contact angle measurements were performed using sessile drop methodology. Three distinct locations were selected per sample for triplicate measurements, with mean contact angles calculated to ±0.1° precision. High-resolution droplet profiles were captured in real-time using integrated optical imaging (1,920 × 1,080 pixel resolution) and analyzed via dedicated image analysis software (Attension Theta v5.0) to quantify wettability parameters, with all raw data archived for precise angle quantification.

### 2.6 Drug release profiling

The drug release kinetics of experimental specimens [Ti-PTL-(HA/HBPL)_3_ and Ti-PTL-HA/HBPL] were evaluated using a standardized elution protocol. Samples were immersed in phosphate-buffered saline (PBS, pH 7.4) within 24-well plates and incubated under gentle agitation (50 rpm) in a thermostatic orbital shaker (37 ± 0.2°C). At predetermined intervals (6 h, 12 h, 1 d, 3 d, 7 d, 10 d, and 14 d), 200 μl aliquots were extracted for quantitative analysis via bicinchoninic acid (BCA) protein assay (Pierce™, Thermo Scientific), with absorbance measured at 562 nm using an M200 Pro microplate reader (Tecan Group Ltd., Switzerland). The release medium was replenished with fresh PBS after each sampling to maintain sink conditions. Cumulative protein release was calculated against a six-point calibration curve (R^2^ > 0.995) and plotted as mean ± SD (*n* = 3 independent replicates).

### 2.7 Agar diffusion antibacterial assay

Test specimens [Ti, PTL, HA/HBPL, and (HA/HBPL)_3_] were precision-cut into discs (10 mm diameter) using laser ablation, sequentially sterilized in absolute ethanol, sonicated in deionized water for 10 min, and vacuum-dried at 80°C for 2 h prior to autoclave sterilization. *Streptococcus gordonii* and *S. sanguinis* were cultured in brain-heart infusion (BHI) broth at 37°C under microaerophilic conditions (5% CO_2_) for 18 h to mid-log phase. Bacterial suspensions were adjusted to 1 × 10^6^ CFU/ml using sterile saline (0.9% NaCl) with optical density calibration at 600 nm (OD_600_ = 0.1). Sterile cotton swabs were used to evenly inoculate Mueller-Hinton agar plates with 100 μl bacterial suspension. Specimens were aseptically placed onto inoculated agar surfaces, ensuring full material-agar contact via sterile forceps. Plates were incubated at 37°C for 48 h under controlled humidity. Post-incubation, inhibition zones were documented using a calibrated imaging system under uniform LED illumination. Colony counting was performed using automated image analysis software (ImageJ v1.53t) with morphological filtering to differentiate inhibition halos from background colonies.

### 2.8 Live/dead bacterial viability assay via confocal laser scanning microscopy

Sterilized specimens were aseptically transferred into 24-well cell culture plates and inoculated with 1 ml of bacterial suspension. Following 24 h incubation at 37°C under 5% CO_2_, the culture medium was aspirated, and specimens underwent three PBS washing cycles with gentle orbital shaking. A fluorescent viability stain cocktail was prepared by mixing SYTO 9 and propidium iodide (PI) at a 1:1 volumetric ratio. Each well received 200 μl of staining solution and was incubated protected from light at ambient temperature for 15 min. Excess dye was removed through three additional PBS rinses under dark conditions.Samples were immediately imaged using a confocal laser scanning microscope. Fluorescence quantification was executed via ZEN 3.4 software, with live bacteria (SYTO 9) and dead bacteria (PI) populations calculated from thresholded binarized images (13).

### 2.9 Scanning electron microscopy analysis of bacterial morphology

The four experimental materials were individually placed in 24-well culture plates and inoculated with 1 ml suspensions of *Streptococcus gordonii* and *S. sanguinis* at a 1:1 ratio (1 × 10^6^ CFU/ml total). Biofilms were cultivated microaerophilically (37°C, 5% CO_2_) for 24 h. Specimens were gently rinsed thrice with PBS under orbital agitation and fixed in 2.5% glutaraldehyde at 4°C for 4 h. A graded ethanol dehydration series (50%, 70%, 80%, 90%, 100% v/v, 15 min per step) was performed using an automated tissue processor. Critical point drying preceded sputter-coating with 5 nm gold-palladium (Quorum Q150T ES, 30 mA, 120 s) to enhance surface conductivity. High-resolution imaging was conducted via field-emission scanning electron microscopy at 5 kV accelerating voltage (14). Bacterial adhesion density, morphological alterations, and spatial distribution patterns were quantified across 10 random fields using SmartSEM v6.0 image analysis suite, with biofilm coverage calculated via threshold-based binarization.

### 2.10 Cell culture

Murine pre-osteoblastic MC3T3-E1 cells were cultured in high-glucose Dulbecco's Modified Eagle Medium (DMEM, Gibco) supplemented with 10% fetal bovine serum (FBS, HyClone) and 1% penicillin/streptomycin (P/S, Sigma-Aldrich) under standard conditions (37°C, 5% CO_2_, 100% humidity). Rat bone marrow-derived mesenchymal stem cells (BMSCs) were maintained in DMEM/F-12 medium (Corning) containing 10% FBS and 1% P/S within a humidified tri-gas incubator (Thermo Scientific Forma Series II) with identical atmospheric parameters.

### 2.11 Lactate dehydrogenase (LDH) cytotoxicity assay on titanium substrates

Confluent MC3T3-E1 cells were trypsinized (0.25% Trypsin-EDTA), centrifuged (300 ×g, 5 min), and resuspended in complete medium to achieve 4 × 10^4^ cells/ml. Sterilized titanium specimens (*n* = 3 technical replicates per group) were aseptically placed in 24-well plates, followed by addition of 1 ml cell suspension per well. Cells were cultured under standard conditions (37°C, 5% CO_2_, 95% humidity) for 24 h.Post-incubation, cells were lysed using RIPA buffersupplemented with protease inhibitors. Lysates were sonicated on ice and centrifuged to obtain clarified supernatants. LDH activity was quantified using the CytoTox 96® Non-Radioactive Cytotoxicity Assay (Promega) according to manufacturer protocols (15). Absorbance at 490 nm was measured with correction at 650 nm using a multimode plate reader.

### 2.12 CCK-8 assay for cellular proliferation on titanium substrates

Cellular proliferation kinetics were assessed daily (Days 1–6) by supplementing cultures with 10% CCK-8 reagent (Dojindo Molecular Technologies) relative to total medium volume. Following 4 h incubation under standard conditions (37°C, 5% CO_2_, 95% humidity), absorbance was quantified at 450 nm (reference wavelength: 650 nm) using a multimode microplate reader. Triplicate technical replicates per group were analyzed at each timepoint, with background subtraction using cell-free control wells.

### 2.13 Alkaline phosphatase activity assay

ALP activity was determined using a microplate-based Alkaline Phosphatase Assay Kit according to the manufacturer's instructions. The optical density was measured at 405 nm using a microplate reader.

### 2.14 Protein adsorption quantification assay

Surface-modified titanium specimens (*n* = 3 technical replicates per group) were aseptically transferred into 24-well plates and incubated with 1 ml DMEM medium supplemented with 10% fetal bovine serum (FBS, HyClone) under standardized culture conditions for 4 h. Following incubation, specimens were gently transferred to fresh plates using sterile forceps and subjected to three PBS washing cycles with orbital shaking (50 rpm, 5 min/wash). Adsorbed proteins were desorbed using 0.5 ml of 1% sodium dodecyl sulfate (SDS, Sigma-Aldrich) solution under constant agitation (200 rpm) at 25 ± 0.5°C for 1 h. Protein concentrations were determined via Pierce™ BCA Protein Assay Kit (Thermo Scientific) following manufacturer protocols, with absorbance measured at 562 nm using a SpectraMax i3x microplate reader.

### 2.15 Alizarin red S staining for mineralized matrix deposition

Cells were fixed with 4% paraformaldehyde (PFA) in PBS (pH 7.4) for 15 min at 25°C. After aspirating the fixative, specimens were triple-rinsed with deionized water. A 0.2% Alizarin Red S (ARS) solution (Sigma-Aldrich, pH 8.3 adjusted with 0.1 M Tris-HCl) was slowly applied to cover cell layers, followed by incubation in dark conditions for 30 min. Excess dye was removed via brief rinsing (3 × 10 s) with deionized water to prevent calcium mineral dissolution. Stained specimens were imaged under phase-contrast microscopy with consistent illumination parameters.

### 2.16 RT-qPCR

Total cellular RNA was extracted using chloroform-based phase separation, followed by reverse transcription into complementary DNA (cDNA) using the PrimeScript™ RT Reagent Kit according to the manufacturer's protocol. Quantitative real-time PCR amplification of target genes and endogenous controls was performed with SYBR™ Premix Ex Taq™ on a StepOnePlus™ Real-Time PCR System (Applied Biosystems). Primer sequences for the target and reference genes are listed below:

OCN: F: TTGGCTTCTGACTGGGTGTC, R: GCCGGAGTCTGTTCACTACC;
OPN: F: GCAAACAAGAGGCCCATTTCA, R: GGACATCGACTGTAGGGACG;
RUNX2: F:GCCTCACTTAGCTAGGTCTCAG,R: AGGGAGGGCTGAACAATGTC;
COLI: F:ACATACAGCTTGTGAGGTCGC,R:CGTGACCACGGGCATAATTC;
GAPDH:F:GGGAGACAGCTCATGCATTTC,R:AATGACTATCCTTGTCCCAAGTCA.

### 2.17 Protein isolation and western blot analysis

Proteins were extracted in RIPA lysis buffer (CW2333s, CWBIO, Shanghai) supplemented with Phosphatase Inhibitor Cocktail (CW2200S, CWBIO, Shanghai) and Phenylmethylsulfonyl fluoride (PMSF) (Sigma). Then, centrifugation at 12,000 rpm for 30 min was performed to remove cell fragments. After centrifugation, protein concentrations of the supernatant were determined with the Bradford assay and the extract boiled in Laemmli buffer. After quantified, protein samples (10–20 μg) were separated by 10% SDS–PAGE for electrophoresis and then transferred to PVDF membranes. After blocking with 5% skimmed milk for 1 h, the membranes were incubated with indicated primary antibodies overnight at 4°C. Then, after washing with TBST for three times, the membranes were incubated with horseradish peroxidase (HRP)-conjugated secondary antibodies (Weiao Biological Company, China) at room temperature for 1 h. Protein bands were visualized using enhanced chemiluminescence (ECL) reagent (Cat:AP34L015-B, Life-iLab, Shanghai) with the Tanon 5,200 imaging system. The band intensities were analyzed by ImageJ (version 1.52a, NIH).

### 2.18 *In vivo* foreign body implantation study

This investigation utilized 12-week-old male Sprague-Dawley rats (Beijing HFK Bioscience Co., Ltd.), with all procedures strictly adhering to the National Institutes of Health (NIH) Guidelines for the Care and Use of Laboratory Animals. The protocol was approved by the Animal Ethics Committee of Tianjin Fifth Central Hospital (AEWC-2023-018). Animals were housed in individually ventilated cages under controlled environmental conditions (25°C, 55% humidity, 12 h light/dark cycle) with *ad libitum* access to autoclaved water and feed.

Bone marrow-derived mesenchymal stem cells (BMSCs) were cultured on HA/HBPL_3_ and bare titanium (Blank Ti) substrates for 7 days, with cellular morphology, distribution, and adhesion characterized via phase-contrast microscopy (Nikon Eclipse Ts2, 20×). Following intraperitoneal anesthesia (pentobarbital sodium, 50 mg/kg), a 0.5 cm longitudinal incision was aseptically created 2 cm caudal to the auricular region. A subcutaneous pocket was surgically prepared using blunt dissection, into which pre-sterilized titanium specimens (*n* = 8 rats/group) were implanted. Wound closure was achieved with 5-0 polypropylene sutures, and postoperative analgesia (meloxicam, 1 mg/kg) was administered for 72 h.

### 2.19 Hematoxylin-eosin (H&E) morphological staining

At 4 and 8 weeks post-implantation, dorsal tissue specimens containing titanium implants and surrounding tissues were harvested. Samples were fixed in 4% neutral buffered formalin (Sigma-Aldrich) for 48 h, followed by dehydration in graded ethanol series (70% to 100%), xylene clearing, and paraffin embedding (Leica EG1160). Serial sections (5 μm thickness) were prepared using a rotary microtome (Leica RM2235).

Deparaffinization was performed through sequential immersion in xylene (3 × 5 min) and rehydration via graded ethanol (100% to 70%). Tissue sections were stained using a commercial H&E kit (Abcam, ab245880) according to manufacturer protocols: hematoxylin incubation (5 min), differentiation in 1% acid alcohol (30 s), bluing in 0.2% ammonium hydroxide (1 min), and eosin counterstaining (2 min). Stained slides were dehydrated (95% and 100% ethanol, 2 min each), cleared in xylene, and mounted with neutral balsam (Sigma-Aldrich).

Histological evaluation was conducted under bright-field microscopy at 100× and 400× magnifications. Morphometric analysis of tissue integration and inflammatory response was performed using NIS-Elements BR 5.30 software, with semi-quantitative scoring based on ISO 10993-6:2016 criteria (8 animals/group).

### 2.20 Statistical analysis

Data collected from at least triplicate experiments are presented as means ± standard deviation (SD) and analyzed with GraphPad Prism 9.0.0 via the one-way analysis of variance (ANOVA). The confidence levels were set as 95% (p* < 0.05), 99% (p** < 0.01), (p*** < 0.001) and (p**** < 0.0001).

## 3 Results

### 3.1 Surface modification of titanium and fabrication/characterization of hyperbranched polylysine (HBPL) self-assembled coatings

Zeta potential measurements revealed that HA exhibited a surface charge of −52.47 ± 1.02 mV at pH 7, indicating strong negative charge density conducive to colloidal stability in aqueous solutions. Conversely, HBPL demonstrated a positive zeta potential of +26.80 ± 0.87 mV, attributed to protonated amine groups within its dendritic architecture, which enhanced its dispersibility and interfacial stability (Figure 1a). The complementary electrostatic profiles of HA and HBPL facilitated the formation of stable polyelectrolyte complexes via charge-driven self-assembly. Congo red staining, which selectively binds β-sheet-rich protein conformations, uniformly colored PTL-modified titanium surfaces (Figure 1b), confirming the retention of amyloid-like structural motifs. Untreated titanium substrates displayed smooth, metallic surfaces with characteristic silver-gray luster. PTL functionalization introduced an opaque white proteinaceous layer, while subsequent HA treatment yielded a duller appearance with reduced reflectivity, indicative of surface charge modulation for electrostatic assembly. Sequential HBPL deposition and cyclic coating procedures produced darker hues without macroscopic morphological alterations, suggesting progressive optimization of coating thickness and homogeneity (Figure 1d).

**Figure 2:**
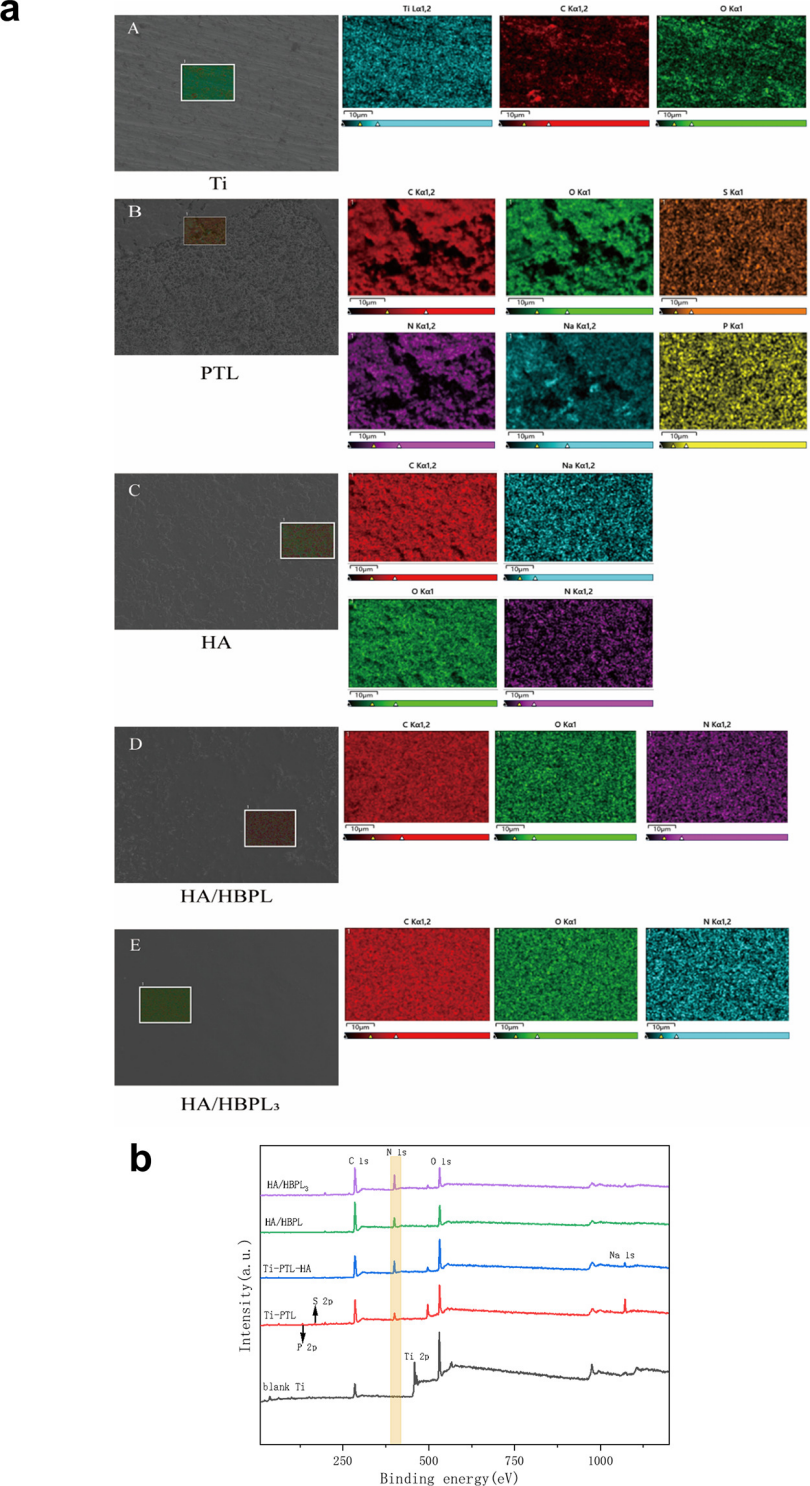
**(a)** EDS elemental mapping of specimens at different modification stages and after self-assembly. **(b)** XPS survey spectra of specimens at different modification stages and after self-assembly.

Scanning electron microscopy (SEM) elucidated the morphological evolution of titanium surfaces under distinct modification protocols (Figure 1e). The Ti-PTL surface exhibited uniformly distributed globular nanostructures, while HA deposition yielded a smoother topography compared to pristine PTL-modified surfaces. Multilayered LbL-assembled coatings [Ti-PTL-(HA/HB)_3_] demonstrated enhanced surface homogeneity and reduced roughness relative to monolayer counterparts [Ti-PTL-(HA/HB)], indicative of progressive structural optimization.XPS survey spectra revealed elemental distribution profiles across modified surfaces (Figures 2a,b), calibrated using the adventitious carbon C 1s peak (284.8 eV). Untreated titanium displayed characteristic Ti 2p_3_/_2_ (458.4 eV) and O 1s (532 eV) peaks. PTL-functionalized surfaces exhibited emergent S 2p (168.2 eV) and P 2p (132.2 eV) signatures originating from tris(2-carboxyethyl)phosphine (TCEP), concurrent with the disappearance of Ti 2p_3_/_2_ signals, confirming complete surface coverage by phase-transitioned lysozyme. Subsequent HA deposition introduced a Na 1s peak (1,071 eV), verifying successful anionic polymer immobilization. Layer-by-layer HBPL assembly generated a prominent N 1s peak (400 eV), attributable to amine and amide functionalities, validating cationic polyelectrolyte incorporation. These results convincingly demonstrate the feasibility of constructing stable LbL architectures on titanium substrates, culminating in the successful fabrication of self-assembled Ti-[HA/HBPL]_3_ antimicrobial coatings.

**Figure 3:**
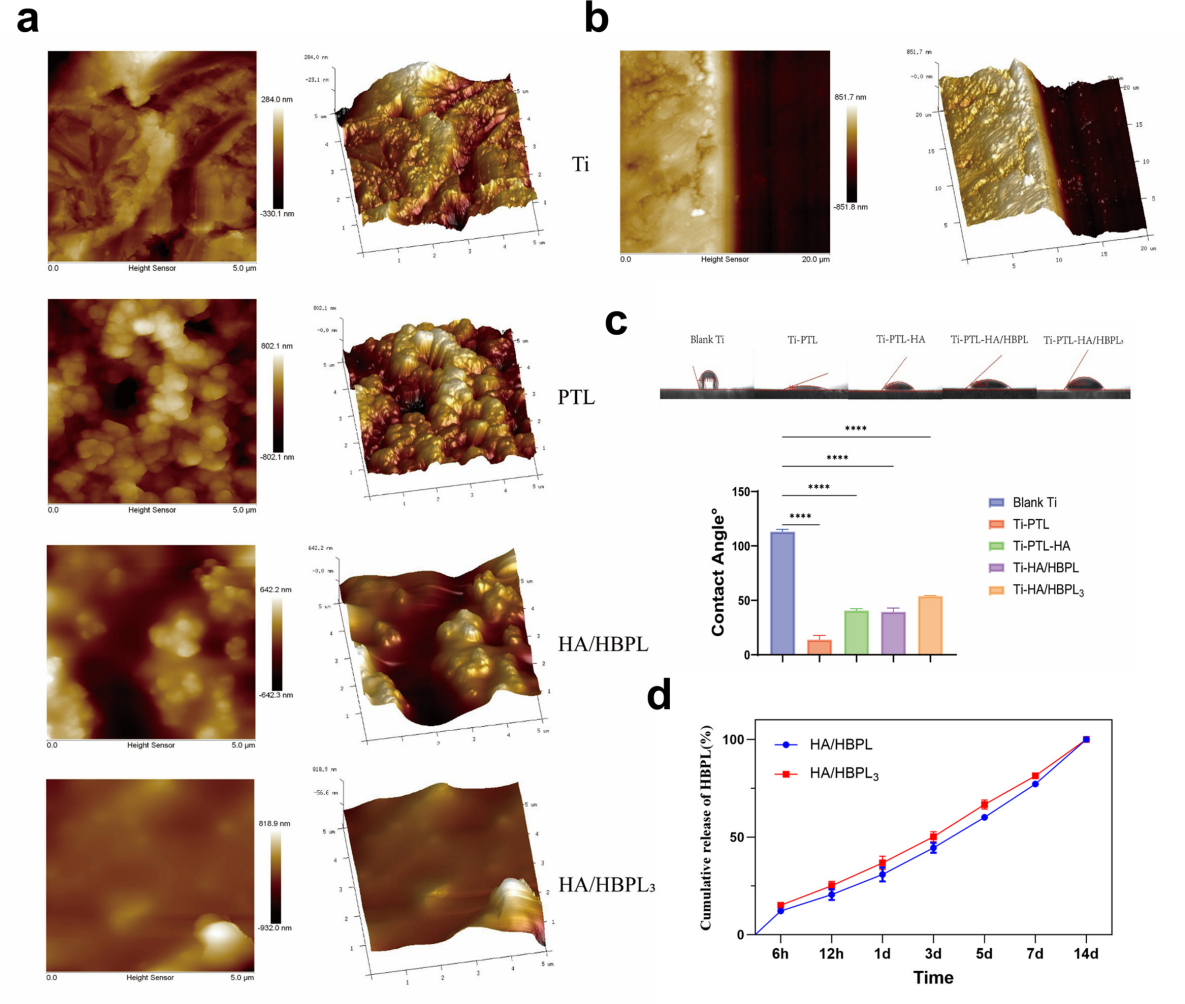
**(a)** AFM images of different material surfaces. **(b)** AFM image showing the thickness of HA/HBPL_3_ coating. **(c)** Water contact angles on various specimen surfaces. **(d)** Drug release profiles of polyelectrolyte-modified coatings. ****P < 0.0001.

Atomic force microscopy (AFM) revealed distinct surface topographies across modified titanium groups (Figure 3a). The Ti-PTL group exhibited significantly increased surface roughness compared to pristine titanium (Ra: 85.3 ± 12.1 nm vs. 22.4 ± 3.5 nm). Subsequent LbL assembly of HA and HBPL reduced surface roughness (Ra: 48.7 ± 6.9 nm), effectively masking the PTL underlayer. Progressive LbL cycles further optimized coating uniformity, culminating in a smooth, densely packed multilayer structure with a total thickness of 1.0 ± 0.1 μm (Figure 3b). Water contact angle measurements demonstrated dynamic wettability evolution during coating fabrication (Figure 3c). Untreated titanium exhibited inherent hydrophobicity (113.18 ± 1.02°), while PTL functionalization dramatically enhanced hydrophilicity (13.97 ± 3.80°), attributable to surface enrichment of polar groups (amino, hydroxyl, carboxyl) from denatured lysozyme. HA deposition moderately increased contact angles to 40.77 ± 1.58°, likely due to steric reorganization of hydrophilic domains. Subsequent HBPL assembly stabilized wettability (39.6 ± 3.32°), with terminal amine groups compensating for HA's spatial effects. Final LbL-optimized coatings [(HA/HBPL)_3_] maintained balanced hydrophilicity (54.00 ± 0.44°), representing a 52% improvement over untreated titanium.

Additionally, the cumulative release of HBPL from the modified titanium in PBS solution exhibited a time-dependent increase in both experimental groups, characterized by an initial rapid release phase followed by gradual attenuation (Figure 3d).

### 3.2 Antibacterial properties of modified titanium surface

**Figure 4:**
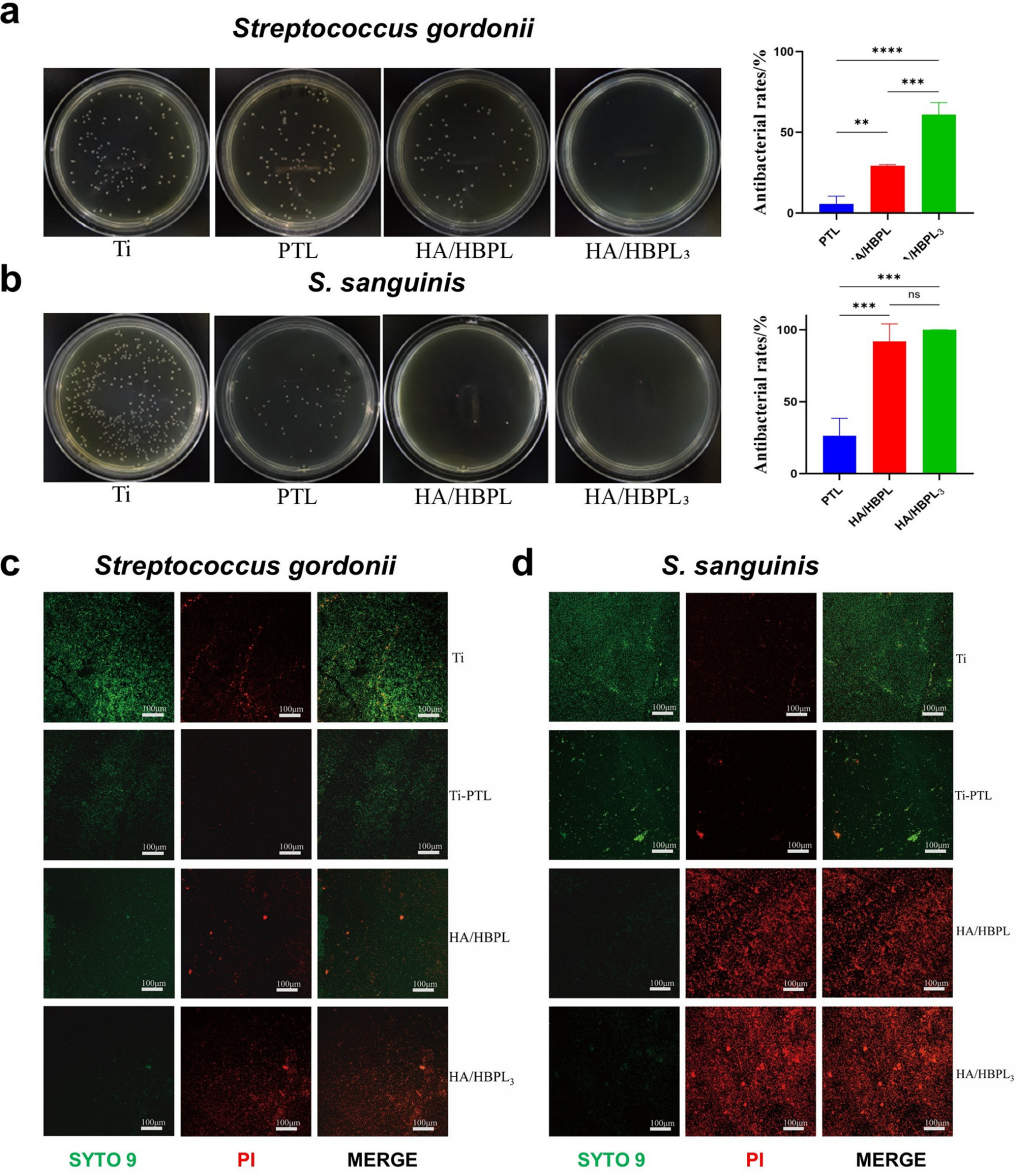
**(a)** antibacterial performance of HA/HBPL_3_ against *Streptococcus gordonii* and quantitative analysis results. **(b)** Antibacterial performance of HA/HBPL_3_ against *S. sanguinis* and quantitative analysis results. **(c)** Confocal imaging comparisons of *S. gordonii* colonization patterns. **(d)** Confocal imaging comparisons of *S. sanguinis* colonization patterns. ns, *P* > 0.05; **: *P* < 0.01; ***: *P* < 0.001; ****: *P* < 0.0001.

The agar diffusion assay revealed distinct bactericidal effects across experimental groups. Pure Ti substrates demonstrated the highest colonization density for both *Streptococcus gordonii* and *S. sanguinis*, with confluent biofilm formation nearly covering the entire agar surface (Figures 4a,b). PTL-modified surfaces exhibited a moderate reduction in colony counts (approximately 30% decrease vs. Ti), though bacterial distribution remained uniform. HA/HBPL coatings significantly suppressed *S. gordonii* growth (70% reduction) while nearly eliminating *S. sanguinis* colonization (99% inhibition). Remarkably, the HA/HBPL_3_ group showed near-complete bactericidal efficacy (99.93 ± 0.09% inhibition), with only sporadic colonies observed.

Live/dead fluorescence imaging corroborated these findings (Figures 4c,d). Pure Ti surfaces displayed dense populations of viable bacteria. PTL modification reduced viable counts by 40%, though bacterial vitality remained high. HA/HBPL surfaces exhibited membrane-compromised *S. gordonii* and near-complete eradication of *S. sanguinis*. The HA/HBPL_3_ group demonstrated exceptional antimicrobial performance, with minimal viable bacteria detected (<1% survival).

**Figure 5:**
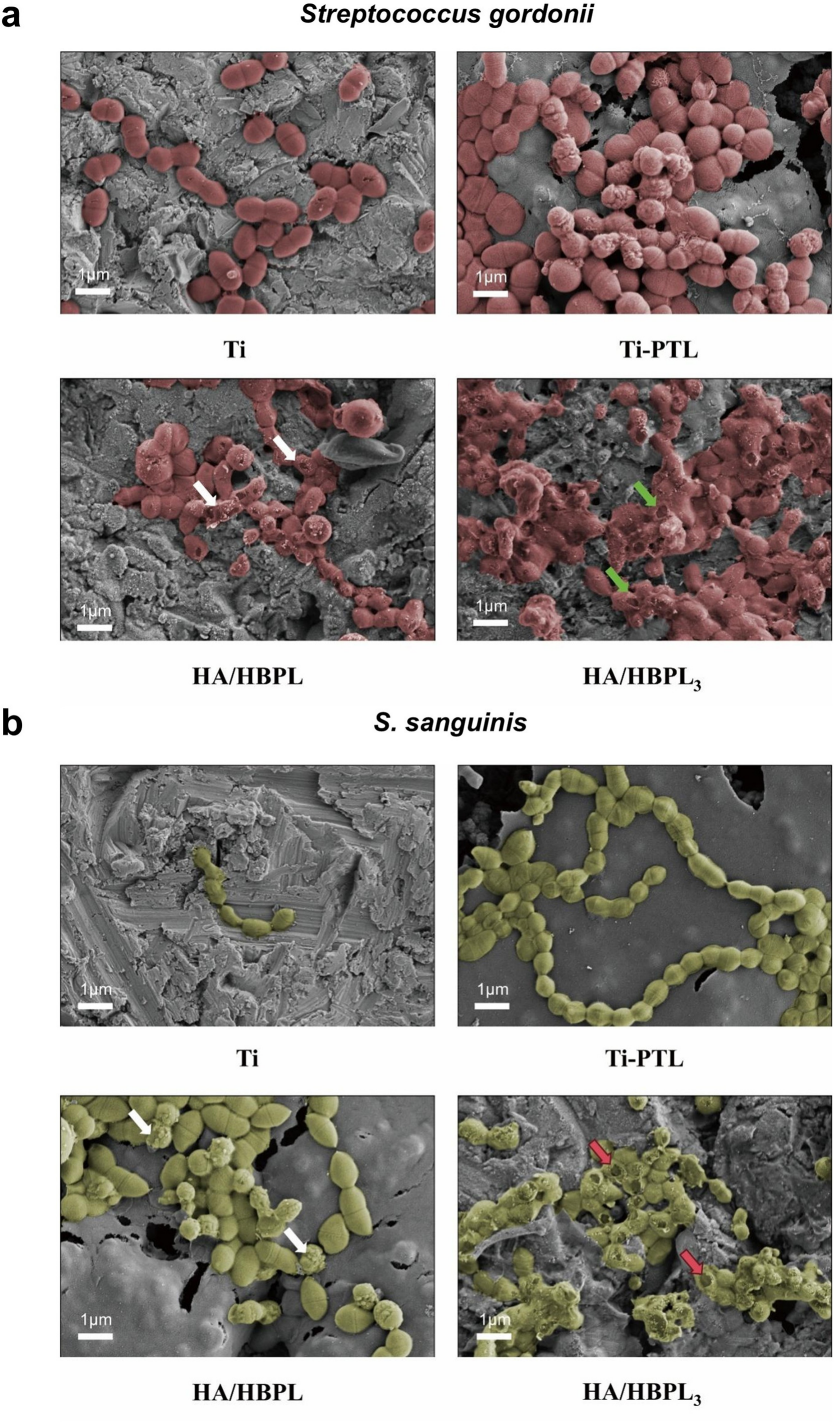
**(a)** FE-SEM images of *Streptococcus gordonii*. **(b)** FE-SEM images of *S. sanguinis*.

High-resolution SEM imaging revealed mechanistic insights (Figures 5a,b). Pure Ti surfaces hosted intact *S. gordonii* (red pseudocolor) with preserved cellular morphology and dense colonization. HA/HBPL surfaces showed reduced adhesion density and partial membrane disruption (white arrows). HA/HBPL_3_ coatings induced catastrophic membrane damage (green arrows), characterized by cytoplasmic leakage and cell lysis. Similar trends were observed for *S. sanguinis* (green pseudocolor), with HA/HBPL_3_ surfaces achieving complete membrane disintegration (white/red arrows).

### 3.3 Biocompatibility and *in vivo* heterotopic osteogenesis of modified titanium

**Figure 6:**
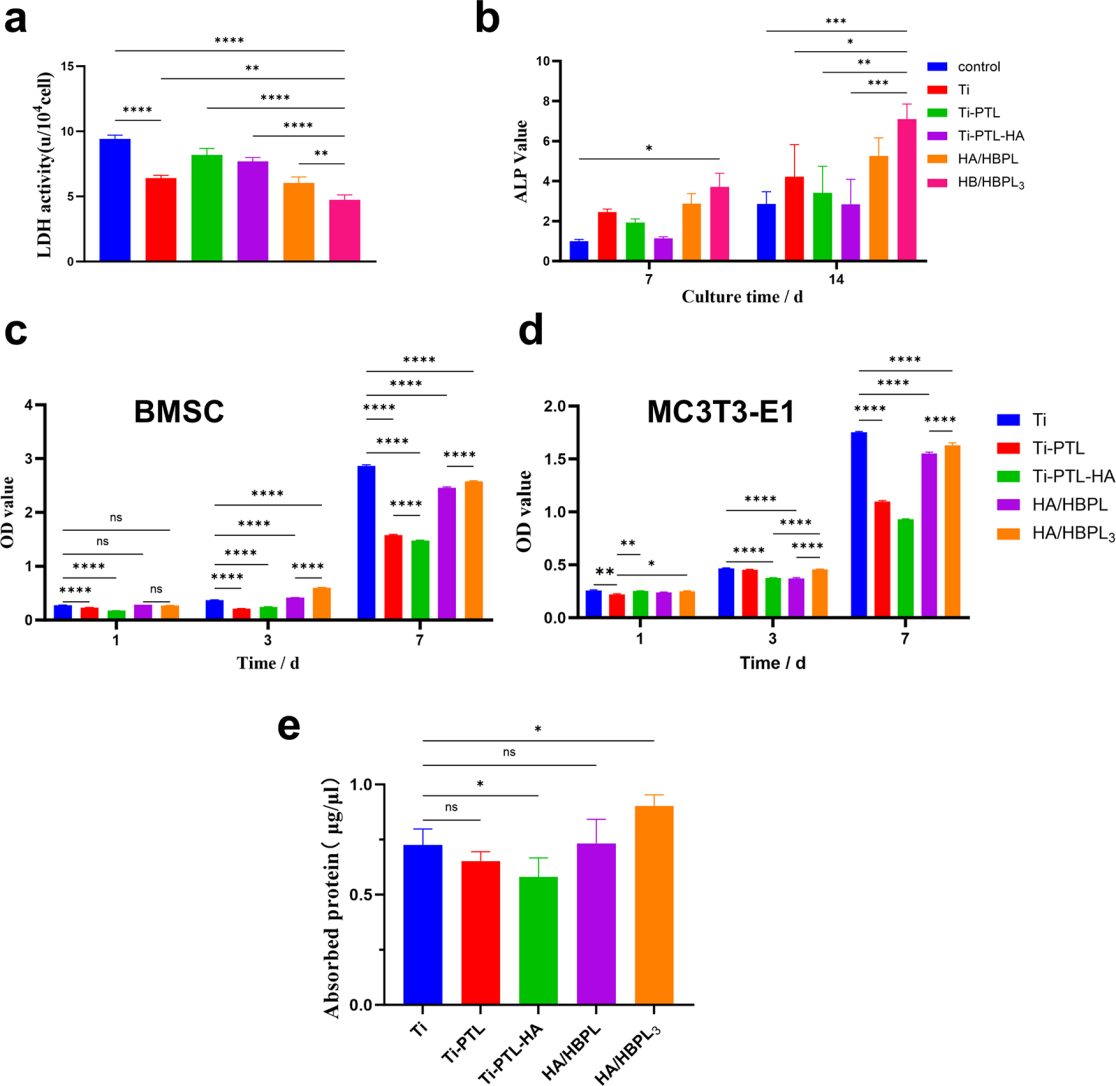
**(a)** LDH activity changes. **(b)** ALP activity changes. **(c)** BMSC proliferation assessment on differently treated titanium surfaces. **(d)** MC3T3-E1 proliferation assessment on differently treated titanium surfaces. **(e)** Protein adsorption quantification. ns: *P* > 0.05; *: *P* < 0.05; **: *P* < 0.01; ***: *P* < 0.001; ****: *P* < 0.0001.

The HA/HBPL_3_ group demonstrated the lowest lactate dehydrogenase (LDH) activity, showing statistically significant differences compared to the Ti group. A sequential reduction in LDH activity was observed across Ti-PTL, Ti-PTL-HA, HA/HBPL, and HA/HBPL_3_ groups. Notably, Ti-PTL and Ti-PTL-HA exhibited elevated LDH activity relative to pure Ti (Figure 6a), suggesting transient metabolic stress during early-stage cell adaptation. These results confirm the minimal cytotoxicity of the composite coatings.

To evaluate osteogenic differentiation, ALP activity was quantified at days 7 and 14. The HA/HBPL_3_ group showed significantly enhanced ALP activity at both timepoints (2.3-fold increase vs. Ti at day 14), indicating sustained promotion of osteoblastic differentiation (Figure 6b).

Proliferation kinetics of rat bone marrow-derived mesenchymal stem cells (BMSCs) and murine pre-osteoblastic MC3T3-E1 cells were assessed. For BMSCs, the HA/HBPL_3_ group exhibited higher optical density (OD) values than Ti at day 3, with a slight reduction by day 7. Ti-PTL and Ti-PTL-HA showed no significant impact on BMSC proliferation (Figure 6c). In contrast, HA/HBPL_3_ significantly enhanced MC3T3-E1 proliferation at both days 3 and 7 compared to HA/HBPL and Ti-PTL-HA groups (*p* < 0.01, Figure 6d), demonstrating cell lineage-dependent bioactivity.

Protein adsorption capacity, indicative of surface bioactivity, was highest in the HA/HBPL_3_ group (2.8 ± 0.3 μg/cm^2^), significantly exceeding Ti (1.1 ± 0.2 μg/cm^2^) and HA/HBPL (1.9 ± 0.2 μg/cm^2^). Ti-PTL-HA showed reduced adsorption (0.8 ± 0.1 μg/cm^2^) compared to Ti, while Ti-PTL exhibited comparable levels to Ti (1.0 ± 0.2 μg/cm^2^) (Figure 6e).

**Figure 7:**
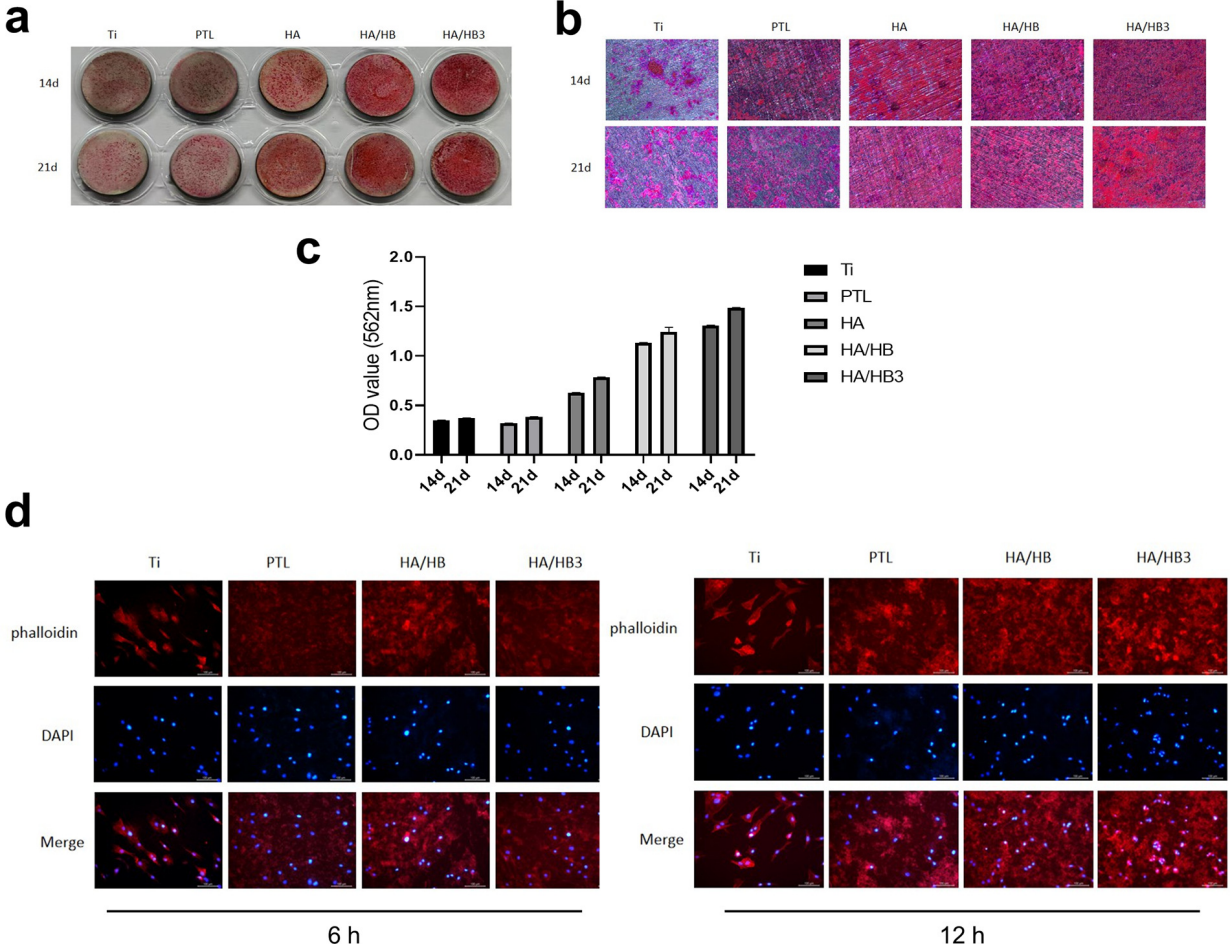
**(a)** macroscopic view of alizarin Red S staining. **(b)** Microscopic observation of Alizarin Red S-stained specimens. **(c)** Quantitative analysis of Alizarin Red S staining intensity. **(d)** Cellular responses and morphological changes at 6 h and 12 h post-treatment.

Alizarin Red S staining demonstrated time-dependent mineralization capacity across groups (Figures 7a–c). The Ti group exhibited faint red staining, indicative of minimal calcium deposition. Gradual intensification of staining was observed in PTL, HA, and HA/HBPL groups, with the HA/HBPL_3_ group showing the most robust red coloration at both 14 and 21 days. HA/HBPL_3_ substrates displayed densely distributed, uniform mineralized nodules with 2.3-fold higher calcium content compared to Ti, confirming enhanced osteogenic mineralization.

Phalloidin/DAPI staining revealed superior cellular integrity on HA/HBPL_3_ surfaces (Figure 7d). At 6 and 12 h post-seeding, HA/HBPL_3_-supported cells exhibited well-defined actin filaments and organized nuclear architecture, with a 40% increase in cell spreading area vs. Ti. In contrast, Ti, PTL, and HA/HBPL groups showed dispersed cytoskeletal networks and irregular nuclear arrangements, suggesting HA/HBPL_3_'s protective effects on cellular homeostasis.

**Figure 8:**
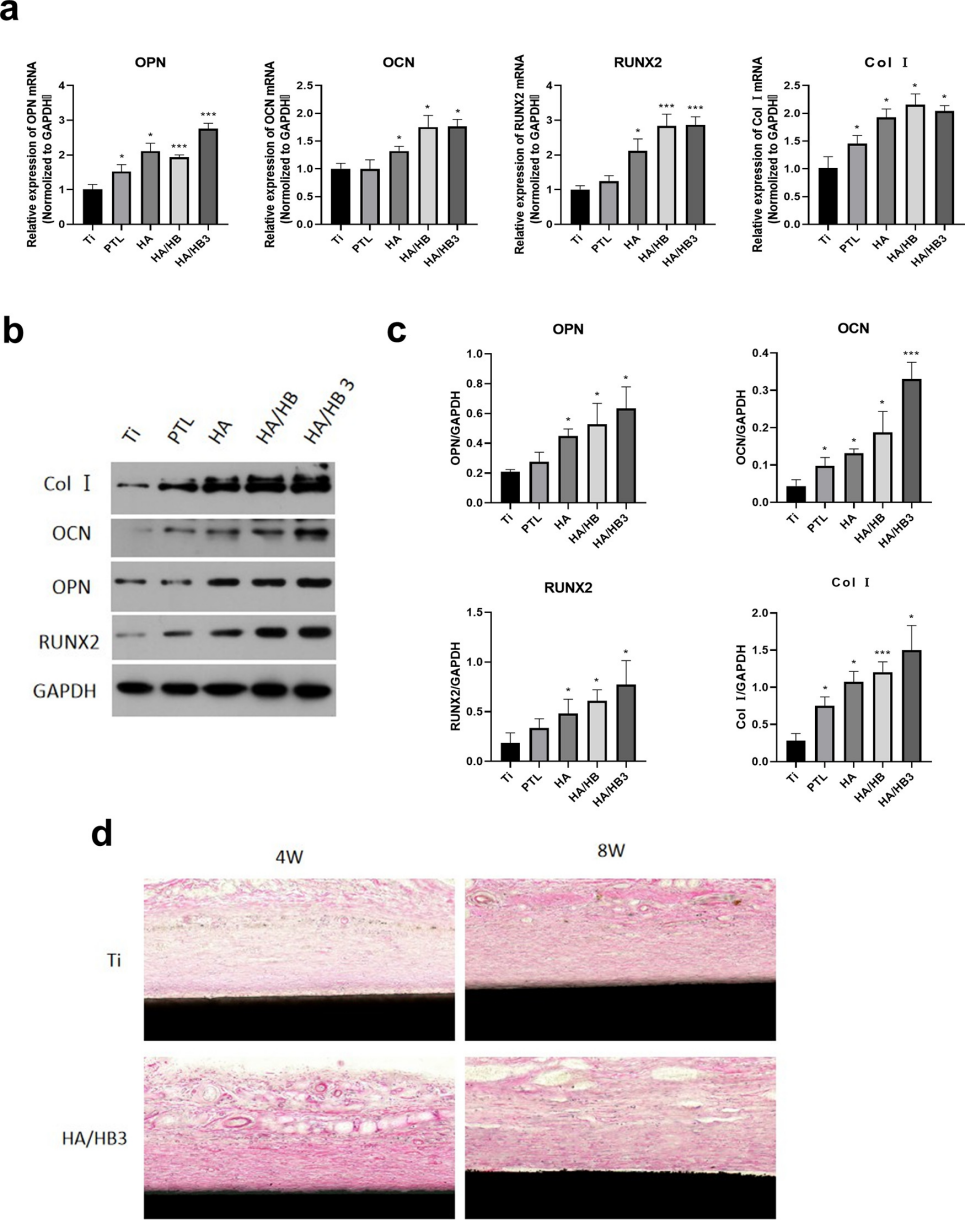
**(a)** expression of osteogenic-related genes. **(b)** Expression of osteogenic-related proteins. **(c)** Quantitative analysis of osteogenic-related protein expression. **(d)** H&E staining of hard tissue sections from ectopic implantation. *P < 0.05; ***P < 0.001.

To evaluate the effects of different treatment conditions on bone metabolism-related genes, the expression levels of COL I, OPN, OCN, and RUNX2 genes were detected using RT-qPCR under different treatment conditions. The results showed that the HA/HBPL3 group exhibited better expression effects in all tested genes, significantly higher than the Ti group, particularly in the expression of OPN and RUNX2, which showed significant increases. This indicates that the HA/HBPL3 treatment significantly promotes the differentiation and bone matrix synthesis of mesenchymal stem cells, potentially through the activation of related signaling pathways, thereby enhancing bone formation. In contrast, the expression levels of the genes in the Ti, PTL, HA, and HA/HBPL groups were relatively stable. Therefore, the HA/HBPL3 group may have a stronger potential in promoting bone generation (Figure 8a). The protein expression levels of COL I, OPN, OCN, and RUNX2 were also detected using WB technology in different treatment groups. The results showed that the HA/HBPL3 group exhibited significant upregulation in all target proteins, particularly COL I and OCN, whose expression levels were significantly higher than those in other groups. Additionally, OPN and RUNX2 proteins in the HA/HBPL3 group also showed increases, indicating that this group of treatment conditions exhibited the strongest effects in promoting the synthesis of related bone proteins. This suggests that the HA/HBPL3 group may enhance the synthesis of bone proteins through certain signaling pathways or molecular mechanisms, thereby promoting the differentiation and maturation of mesenchymal stem cells (Figures 8b,c).

In addition, to comprehensively evaluate the bone-forming ability and biocompatibility of HA/HBPL3, we implanted the modified titanium plates into the back of mice to observe whether HA/HBPL3 could influence tissue healing or promote the formation of anomalous bone tissue under the complex physiological conditions of the body. The results showed that at 4 weeks, fibrous tissue surrounding the HA/HBPL3-modified titanium plates appeared, indicating that HA/HBPL3 could not induce anomalous bone formation without bone scaffold materials or natural bone structures. The wounds of the mice healed well, and no obvious inflammatory cells were observed under the microscope, suggesting that the modified coating had good biocompatibility (Figure 8d).

## 4 Discussion

Surface modification techniques, which effectively endow substrate materials with novel functional properties without altering their inherent structural integrity, have garnered significant attention in materials science. To date, a spectrum of physicochemical methodologies has been extensively applied in surface engineering, including physical adsorption (16), plasma treatment (17), chemical vapor deposition (CVD) (18), and self-assembled monolayers (SAMs) (19). These advanced strategies have established pivotal roles across biomedical applications, ranging from implant biocompatibility enhancement to targeted drug delivery systems, underscoring their transformative potential in modern medical technologies.

In this study, we constructed a multilayered drug delivery system using negatively charged HA and positively charged HBPL, achieving direct drug release via a sacrificial template strategy. HA, a natural polysaccharide, exhibits non-toxicity, film-forming capability, biodegradability, and excellent biocompatibility (20). HBPL, characterized by its non-ordered, highly branched architecture and abundant terminal amino groups, combines structural motifs of both linear poly-L-lysine (PLL) and *ε*-polylysine (*ε*-PL). Recent studies have demonstrated that HBPL-modified titanium surfaces exhibit enhanced osseointegration, broad-spectrum antibacterial efficacy, and anti-inflammatory properties, positioning it as a multifunctional platform for advanced implant applications (21). Leveraging the electrostatic attraction between polyanionic HA and polycationic HBPL, the two components were alternately deposited on titanium surfaces to form polyelectrolyte complex films. Through three cycles of electrostatic adsorption, we successfully fabricated an antimicrobial polyelectrolyte coating with a trilayer “sandwich-like” architecture [(HA/HBPL)_3_]. By adjusting the number of adsorption cycles and initial polyelectrolyte concentrations, the drug release kinetics can be precisely modulated to achieve sustained release profiles. This multilayered structural design enables enhanced drug-loading capacity, combinatorial therapeutic options, and prolonged pharmacological activity duration.

The self-assembled polyelectrolyte films demonstrated structural integrity and stability while preserving their native morphology. Elemental composition analysis of the polyelectrolyte coatings confirmed successful layer deposition through the emergence of characteristic elements and concomitant depletion of substrate-specific signatures from preceding layers. Furthermore, enhanced surface hydrophilicity—a critical factor promoting implant osseointegration—was achieved post-self-assembly, as evidenced by water contact angle assays in this study (22, 23). These findings collectively validate the feasibility of constructing polyelectrolyte multilayers via the LbL technique on PTL-modified titanium substrates, establishing a viable and effective strategy for developing functionalized implant surfaces with tailored physicochemical properties.

In this study, the HA/HBPL coating demonstrated significant antibacterial efficacy against oral pathogenic bacteria *Streptococcus gordonii* and *S. sanguinis*, providing a novel strategy to address bacterial infections and biofilm formation in oral biomaterials (24). Bacterial infections pose a critical challenge in dental implants and restorative materials, particularly due to biofilm development, which not only shields bacteria from antimicrobial agents but also fosters antibiotic resistance and chronic inflammation, ultimately compromising the long-term stability of implants (25). Consequently, the development of antimicrobial materials capable of inhibiting biofilm formation represents a pivotal research focus in the field of oral biomaterials.

Compared to conventional metal ion-based antibacterial approaches (26), the HA/HBPL coating not only demonstrates superior antimicrobial efficacy but also ensures biosafety and biodegradability in clinical applications due to its structural homology with biological matrices. This effectively circumvents potential cytotoxic effects associated with metal ion leaching. Confocal laser scanning microscopy (CLSM) analysis of live/dead stained bacteria revealed extensive red fluorescence across HA/HBPL-coated surfaces, indicating catastrophic membrane integrity loss and cytolysis in bacterial populations. In stark contrast, Ti and PTL substrates exhibited predominantly viable bacteria (green fluorescence), confirming the exceptional antibacterial superiority of the HA/HBPL coating. Antimicrobial peptides (AMPs) typically exert bactericidal effects by binding to bacterial cell membranes, altering membrane permeability, and ultimately inducing membrane disruption through lysis (27). Scanning electron microscopy (SEM) observations further corroborated this mechanism, revealing significant morphological deformations in bacteria adherent to HA/HBPL-coated surfaces, including membrane rupture and structural collapse. These findings demonstrate that the HA/HBPL coating likely compromises bacterial membrane integrity as its primary antimicrobial mode of action.

Although this study selected *S. gordonii* and *S. sanguinis*—two pioneer species in dental plaque formation—for experimental validation, the complexity of the oral microbiome far exceeds interactions between these two bacterial species. Future investigations should incorporate additional representative oral pathogens such as *Porphyromonas gingivalis* (28), *Fusobacterium nucleatum* (29), and even infection-associated *Staphylococcus aureus* to evaluate the broad-spectrum antimicrobial efficacy of the HA/HBPL coating across a comprehensive oral microbial consortium, thereby validating its clinical applicability. Furthermore, while the HA/HBPL coating demonstrated promising short-term antibacterial performance, its long-term stability and durability under physiological conditions require systematic evaluation through accelerated aging tests and simulated oral environment models.

Furthermore, this study systematically evaluated the effects of HA/HBPL_3_ on osteogenic differentiation, proliferation, and bone formation through multiple experimental approaches. The results demonstrated that HA/HBPL_3_ significantly enhanced osteoblast differentiation and matrix mineralization by upregulating osteogenesis-related RNA and protein expression levels, including COL1A1, OPN, OCN, and RUNX2, while concurrently increasing alkaline phosphatase (ALP) activity and promoting cell proliferation without observable cytotoxicity. Alizarin Red S staining, cytoskeletal fluorescence imaging, and protein adsorption assays further corroborated its capacity to facilitate mineralization and strengthen cell-matrix interactions.

In clinical applications, the HA/HBPL_3_ composite coating holds significant promise for addressing bone defects complicated by comorbidities and enhancing dental implant osseointegration (30). By promoting the osteogenic function of osteoblasts, this coating can effectively augment cell-material interactions and provide innovative solutions for implant surface engineering. Furthermore, HA/HBPL_3_ offers a novel therapeutic modality in oral implantation, facilitating the restoration of physiological function and anatomical structure while accelerating healing processes and improving long-term outcomes, thereby enhancing patients' quality of life (31).With growing clinical demands, HA/HBPL_3_ is poised to serve as a critical adjunct to conventional bone grafting biomaterials, particularly in scenarios with limited donor bone availability, offering patients a safe and effective alternative (32). Traditional bone grafting techniques face challenges including donor site morbidity and surgical risks. As a biodegradable material, HA/HBPL_3_ addresses these limitations by eliminating concerns over wear particle-induced foreign body reactions and reducing dependence on technique-sensitive procedures.Future research directions will focus on elucidating HA/HBPL_3_'s capacity to regulate cell signaling pathways and bone metabolism-related gene expression, thereby modulating bone formation processes. This mechanistic exploration will further establish its potential as a next-generation bioactive interface for bone regeneration applications.

As a dual-functional coating exhibiting both antimicrobial activity and osteoinductive potential, HA/HBPL_3_ demonstrated favorable biocompatibility. However, clinical translation necessitates comprehensive validation through long-term efficacy studies, *in vivo* animal trials, and synergistic integration with complementary therapeutic strategies.

## 5 Conclusion

This study developed a dual-functional implant surface coating with combined antibacterial and osteogenic-enhancing properties. The coating was constructed through LbL self-assembly of biomolecules driven by electrostatic interactions, which optimized the physicochemical characteristics of the implant surface. The engineered coating not only exhibited robust antimicrobial efficacy and excellent biocompatibility but also significantly promoted bone regeneration. This innovative strategy provides a novel approach for clinical surface modification of dental implants and offers promising solutions for both preventing and managing peri-implantitis.

## Data Availability

The original contributions presented in the study are included in the article/Supplementary Material, further inquiries can be directed to the corresponding author.
